# Versatile magnetic microdiscs for the radio enhancement and mechanical disruption of glioblastoma cancer cells†

**DOI:** 10.1039/d0ra00164c

**Published:** 2020-02-25

**Authors:** Selma Leulmi Pichot, Sabrina Bentouati, Saif S. Ahmad, Marios Sotiropoulos, Raj Jena, Russell Cowburn

**Affiliations:** Department of Physics, University of Cambridge JJ Thomson Avenue Cambridge CB3 0HE UK sl766@cam.ac.uk; Department of Oncology, University of Cambridge, Hutchison/MRC Research Centre Cambridge Biomedical Campus Cambridge CB2 0XZ UK; Division of Molecular and Clinical Cancer Sciences, School of Medical Sciences, Faculty of Biology, Medicine and Health, The University of Manchester Manchester UK

## Abstract

This study describes the use of highly versatile, lithographically defined magnetic microdiscs. Gold covered magnetic microdiscs are used in both radiosensitizing cancer cells, acting as intracellular emitters of secondary electrons during radiotherapy, and as well as inducing mechanical damage by exerting a mechanical torque when exposed to a rotating magnetic field. This study reveals that lithographically defined microdiscs with a uniform size of 2 microns in diameter highly increase the DNA damage and reduce the glioblastoma colony formation potential compared to conventional radiation therapy. Furthermore, the addition of mechanical disruption mediated by the magnetic component of the discs increased the efficiency of brain cancer cell killing.

## Abstract 2

First study demonstrating the use of physically engineered magnetic particles that display two functionalities for cancer treatment.

## Graphical abstract

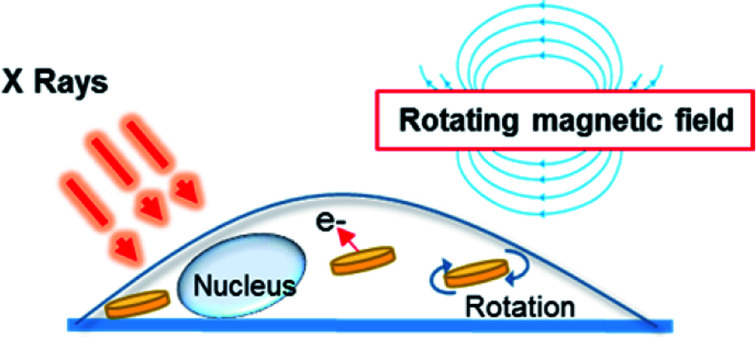

## Introduction

Radiation therapy, which represents the use of ionizing radiation for cancer treatment, is currently widely used to treat primary brain tumors such as glioblastoma. Clinical data demonstrate that radiation therapy prolongs life in patients with glioblastoma, but most patients will develop a recurrence of their disease leading to death within 16–18 months of optimal radiation therapy.^1^ The search for radio enhancing adjuvant therapies that maximize the radiation doses delivered to cancer cells without affecting healthy brain tissue is one of the most active areas of brain tumor research.

Equally actively studied, mechanobiology is a vibrant and rapidly-progressing field, with a growing contribution of nanotechnology to the understanding and treatment of diseases. The recent development of advanced magnetic micro and nanostructures with complex magnetic responses provide powerful tools to explore new approaches in this emerging field.

In the present study, we report the potential use of gold covered magnetic microdiscs that serve both as efficient radiosensitizers for enhanced radiotherapy, and intracellular mechanical actuators for mechanically induced cell damage in glioblastoma. The radioenhancement during radiotherapy is coupled with the intracellular mechanical disruption with rotating magnetic microdiscs when exposed to an external magnetic field. The goal is to maximize the therapeutic effect of versatile magnetic microdiscs *in vitro*, before moving onto animal models of disease. The delivery of the technology described in this paper to the clinical phase will rely on the latest development in cancer treatment such as the local delivery *via* microdialysis catheters coupled with kilovoltage range Intra Operative Radiation Therapy (IORT) techniques.

Advances in physical sciences and nanotechnology provide new tools to explore the radioenhancement with metal-based nanomaterials. Studies on nanomaterials that enhance the therapeutic efficiency of radiotherapy have reported the use of a wide range of materials such as bismuth,^2,3^ gold,^4,5^ platinum,^6^ tantalum^7^ and silver.^8^ Among these studies, gold stands as the ideal choice because of the high-mass energy absorption coefficients, biocompatibility, and easy surface modification. To date, investigations have described the use of simple constructs of gold particles with different shapes^9^ (nanoparticles, nanorods or nanospikes), and with various coating strategies to improve the cellular uptake^4,10^. However, no studies have used multi-layered magnetic structures combining gold and other materials to evaluate their potential in cancer radiotherapy.

A new class of magnetic nanomaterials, in which manufacturing techniques usually used in microchip fabrication, have recently been investigated.^11,12^ This new material class exhibits a high level of functionality without the need for complex external equipment. For example, the magnetic nanostructures can be manipulated, guided, concentrated, rotated, vibrated, assembled or disassembled with low magnetic fields.

The magnetic microdiscs used for this study consist of a synthetic antiferromagnetic stack of thin films of tantalum, platinum, cobalt iron boron alloy and ruthenium. The thickness of each thin film and their order are carefully tuned in order to provide specific magnetic properties, such as avoiding the agglomeration of microdiscs in solution and delivering high forces to biological species once actuated with magnetic fields. These advanced magnetic particles deliver tunable forces, ranging from pN (pico newtons) to a few nN (nano newtons). Previous studies investigated ways to synthesize particles with artificially embedded properties and controllable magnetic behaviors^11,12^. When interfaced with cancer cells, the magnetic particles are actuated with external magnetic fields inducing mechanical stress within cells, leading ultimately to the death of the glioma cells.^13^

Besides their intrinsic magnetic properties, the multi-layered magnetic discs have great potential for radioenhancement. The use of such magnetic microdiscs during radiotherapy maximizes the radiation doses delivered to cancer cells at a very short range, by locally enhancing free radical production. Indeed, the microdiscs are made from high atomic number materials such as gold (*Z* = 79), platinum (*Z* = 78) and tantalum (*Z* = 73). These high *Z* layers absorb X-rays energy and emit scattered X-rays/photons, photoelectrons, Auger electrons and Compton electrons (where the predominant effect depends on the energy of the X-ray beam). The secondary electron emission generates reactive oxygen radicals through the ionization of intracellular water molecules and other elements^14^ These free radicals diffuse *via* chain reactions inside the cells and induce irreversible damage to double stranded DNA^15^ and other organelles.^16^ In this sense, the particles act as radiation enhancing sources within tumors. When combined with the cellular weakening from the mechanical stress caused by the particles, this phenomenon leads to efficient damage, localized to a spatially restricted region, sparing neighboring tissue.

The motivation of such a study is to provide microdiscs which would be inserted into the tumour cavity at the time of surgery, as this avoids the need to design blood brain barrier penetrant constructs for systemic (blood based) delivery. Our proposal is that microdiscs are deposited peri-operatively *via* delivery devices that were already developed for the local application of tumoricidal agents such as convection-enhanced delivery catheters^17^

**Fig. 1:**
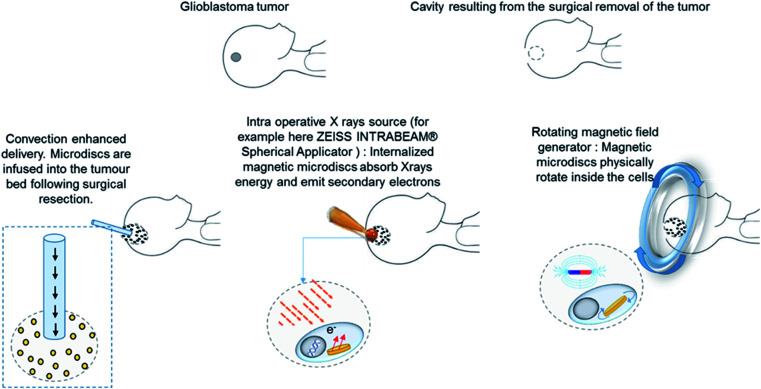
Schematic of the clinical procedure involving the use of magnetic microdiscs for the radioenhancement and mechanical killing of glioblastoma cancer cells.

Rather than using high energy X-rays, we investigated the effect of low energy X rays (195 kV) for the radioenhancement in cells loaded with magnetic microdiscs. With the prospect of using low energy X-ray sources that can be placed into the tumour cavity at the time of surgery. Such intracavitary therapies are already used in the treatment of tumors and are called intra operative radiation therapy. This approach involves the insertion of an X-ray tube at the target site and utilizes low-energy X-ray beams to irradiate locally the cancer cells. This study emphasize the use of microdiscs for intracellular radiation amplification through their intrinsic composition, with an additional leverage of mechanical weakening brought by the magnetic properties. A schematic of the clinical procedure is shown in Fig. 1. The results reported in this paper pave the way to investigate advanced preclinical and clinical studies, conducted both on animal models and on patient derived brain tumor cells. Such studies investigate, along with other parameters, the internalization timescales of the microdiscs in a realistic *in vivo* scenario, allowing the clinician teams to determine the timescales related to the final surgical procedure (intra-operative or post-operative radiotherapy exposure).

In the following sections, we evaluate the cytotoxicity of these microdiscs, as well as their radioenhancement potential on T98G glioblastoma cells. We also report the cellular impact of rotating such magnetic microdiscs located at the intracellular level. We also demonstrate the reduced proliferative activity of the glioblastoma cells using enhanced radiotherapy (RT) using intracellular magnetic microdiscs, and the combination of microdisc-enhanced RT with the mechanical damage using an activating rotating magnetic field.

## Results

### Characterisation of the magnetic construct

The magnetic microdiscs used for this study consist of pairs of perpendicularly magnetized cobalt iron boron (CoFeB) layers coupled by platinum (Pt) and ruthenium (Ru) spacers. This construct prevents the magnetostatic agglomeration of microdiscs thanks to the Ruderman–Kittel–Kasuya–Yosida (RKKY) coupling of the magnetic layers. This configuration creates a state at remanence with no net magnetization as shown on the Vibrating Sample Magnetometer (VSM) measurements in Fig. 1. Furthermore, this particular construct confers a magnetic easy axis on the microdiscs which lies perpendicular to the plane of the particle. For the purpose of applying a mechanical stress to cellular membranes and/or intracellular organelles, the use of particles with an out of plane magnetic anisotropy have been shown to deliver a greater torque when compared to particles with an in-plane anisotropy.^18^ Under a rotating magnetic field strong enough to saturate them along their magnetic easy axis, the magnetic microdisc particles try to rotate in order to keep their magnetization aligned both with the field and their easy axis. In the process, they apply torque to their surroundings which can mechanically damage the cells.

**Fig. 2:**
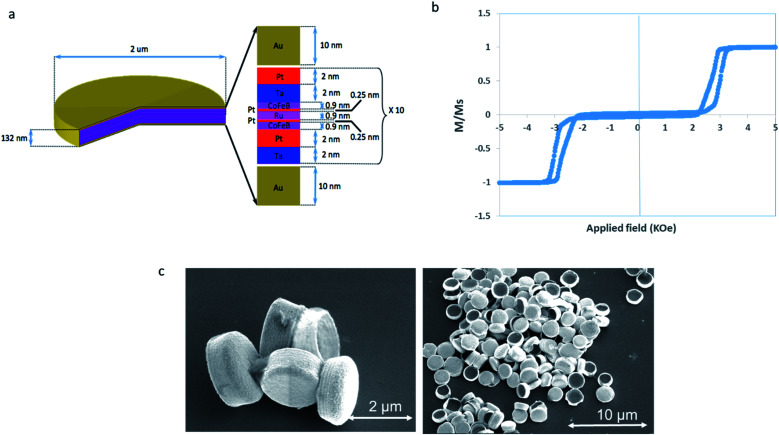
Magnetic microdisc properties. (a) Schematic of the magnetic microdisc composition. (b) VSM hysteresis loop of the multilayer stack. (c) Scanning electron microscopy of released microdiscs.

The building block structure consists of Ta/Pt/CoFeB/Pt/Ru/PT/CoFeB, which is repeated ten times in order to increase the total magnetic moment, giving a total magnetic thickness of 18 nm. This magnetic multilayer stack is grown onto lithographically patterned pillars of MA-N 1410 photoresist. The photoresist pillars are then dissolved in acetone, releasing the 2 micron wide, 132 nm thick discs into solution (Fig. 2). Further details can be found in the Method section.

### Microdiscs internalization

**Fig. 3:**
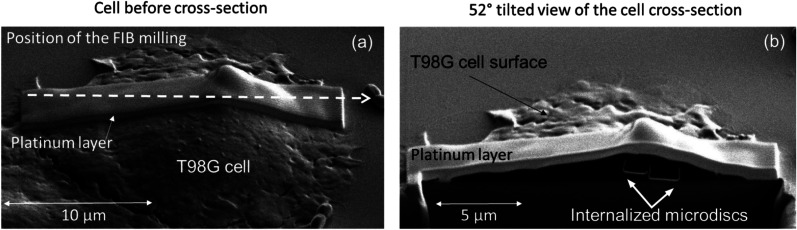
FIB-SEM cross section images of internalized magnetic microdiscs in T98G cells. (a) SEM image before the FIB milling, the white arrow indicates the position of the cross-section seen in image (b). (b) SEM image after the FIB cross section revealing two intracellular microdiscs.

The location of the magnetic microdiscs inside T98G cells was assessed using focused ion beam – scanning electron (FIB-SEM) based tomography. After their fixation and metallization, T98G cells incubated 24 h with magnetic microdiscs are randomly selected and then milled using the ion beam and imaged using the electron beam creating a series of images of the interior of the cell. Internalized magnetic microdiscs are shown in Fig. 3. Similar observation was previously reported by Cheng *et al.*^19^ using similar particles on glioblastoma U87 cell line. Additional FIB-SEM images of internalized microdiscs are shown on the ESI section.†

### Cytotoxicity evaluation

**Fig. 4:**
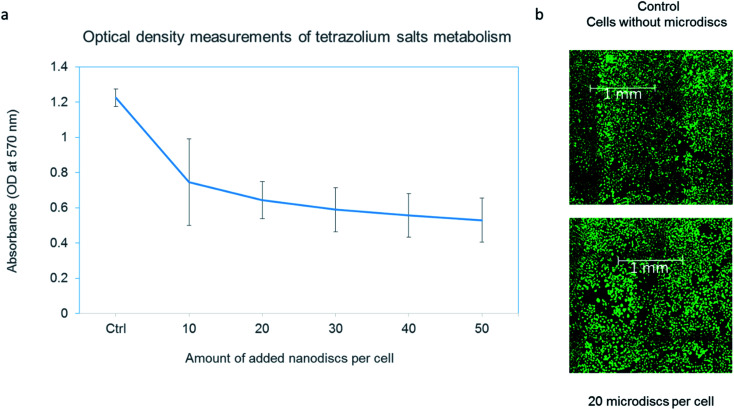
Cytotoxicity assay for microdiscs-loaded cells after 24 h incubation time. (a) Metabolic activity measurement for cells loaded with various microdiscs concentrations. (b) Calcein fluorescence images of control cells and cells loaded with 20 microdiscs per cells. Similar pattern was observed for other microdiscs concentrations displayed on the graph (a).

To ensure that the internalization of the microdiscs does not cause cell damage in itself, a cytotoxicity evaluation test was performed. Cell metabolic ability was examined by MTT assay (Fig. 2(a)). T98G cells were incubated with different concentrations ranging from 10 microdiscs per cell to 50 microdiscs per cell. Cells loaded with particles were compared to control cells. T98G cells loaded with microdiscs exhibited a decreased metabolism of tetrazolium salts in a dose-dependent manner. However, it is important to note that the internalized microdiscs did not affect the cells' viability. Live/Dead assay results suggest that the cells remain viable despite exposure to concentrations up to 50 particles per cell. The cellular density was similar to that in the control well as shown in Fig. 4(b). One can conclude that the internalization of 2 μm wide magnetic discs into the intracellular compartment reduces the metabolic activity of T98G glioma cells. This is an interesting phenomenon for radioenhancement, since cells with a lower metabolism and cell cycle arrest might be an attractive target. As shown on ESI,† the same trend was observed after an incubation time of 24 h, 48 h and 72 h. Although microdisc loaded cells maintained their proliferation activity up to 3 days, a more comprehensive study would enable to assess the long term effect of internalized magnetic microdiscs. Of note, the presence of the microdiscs did not have an effect on the doubling time in the time frame of our experiment (up to 72 h). We confirmed that the exposure of cells to the magnetic field without microdiscs did not show any influence on the metabolic activity.

### Mechanical damage

**Fig. 5:**
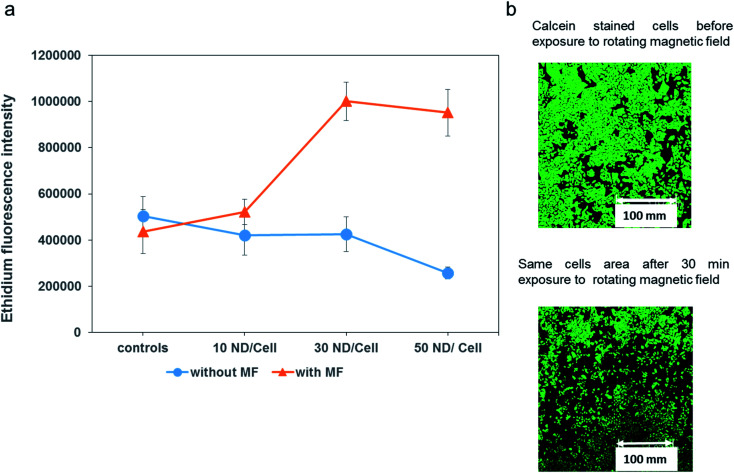
(a) Ethidium bromide fluorescence quantification revealing the extent of cellular damage after 30 minutes exposure to 1 Tesla rotating magnetic field (MF). Controls include cells loaded with same doses of magnetic particles, but non-exposed to the magnetic field. (b) Fluorescence microscopy images of calcein stained cells before and after the exposure to the magnetic field.

T98G cells were incubated 24 h with magnetic microdiscs at different concentrations (see Methods section for details). After this incubation time, microdiscs are internalized by the cells without the need of any particular functionalization to facilitate receptor mediated endocytosis. As shown in Fig. 3, cells loaded with the microdiscs were then exposed to a 1 Tesla rotating magnetic field for 20 minutes. Analysis after this treatment with Live/Dead staining revealed a marked increase in nuclear staining of cells with compromised cell membranes. Representative images of this experiment are shown in Fig. 5. Moreover, an overall decrease in cell population was observed following the treatment. Indeed, while all the conditions had the same initial cell concentration, fluorescence microscopy images after calcein staining revealed areas devoid of cells following the treatment. Such an observation is indicative of a high level of cell injury following intracellular mechanical damage. A fluorescence microscopy image of an area of cells before and after the treatment highlighting this high level of cellular damage is shown in Fig. 5. The control group of cells are for field treatment and particle incubation: we considered cells with an applied magnetic field but no microdiscs, cells with microdiscs but no applied field and cells with neither applied field nor microdiscs. All showed negligible levels of cell death.

### Radioenhancement

**Fig. 6:**
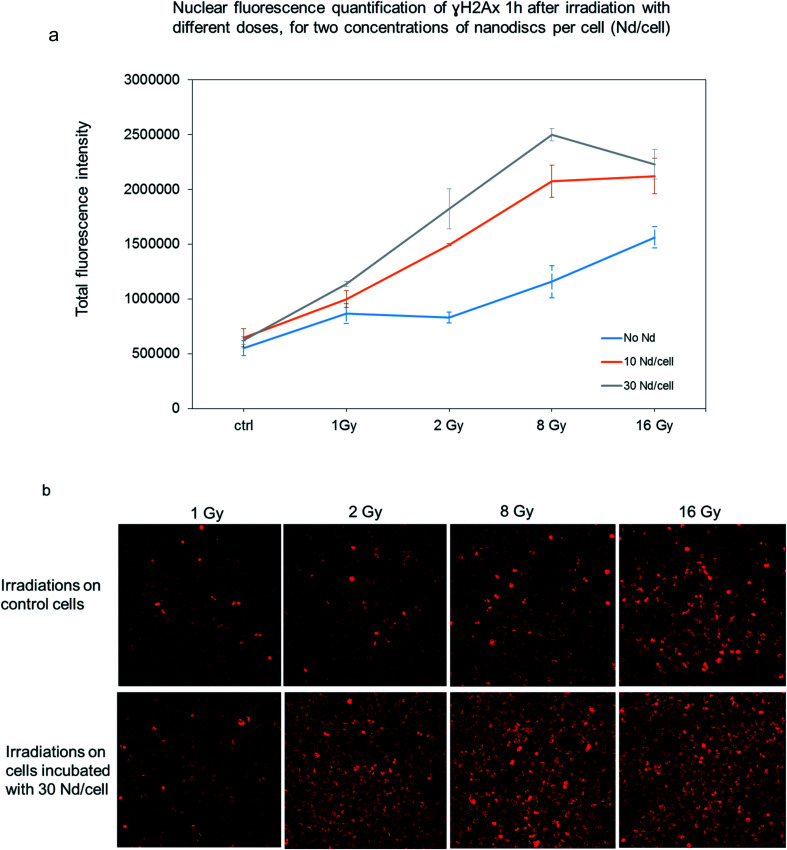
Nuclear fluorescence quantification of γH2AX, 1 hour after irradiation. (a) Total internal fluorescence quantification using Image J software. (b) Confocal fluorescence microscopy images of T98G cells nuclei revealing the γH2AX staining. Radiations doses are expressed in gray (Gy).

To further examine the effect of the radioenhancement mediated by the magnetic microdiscs, we targeted the γH2AX as a marker to reveal DNA damage. In response to DNA double-strand breaks, histone H2AX, is phosphorylated to form γH2AX. At 1 hour post-irradiation, cells loaded with the microdiscs and treated with various doses of radiation displayed an increased intensity of γH2AX foci staining compared to cells treated with equivalent radiation doses as shown in Fig. 6. Rather than counting individual foci, we preferred the quantification of total internal fluorescence for various areas of treated cells for two reasons. First, the microdiscs distribution is not uniform within the entire cell population (cells internalize random numbers of microdiscs). Second, the high level of nuclear damage causes overlapping of multiple foci which prevent the accurate counting of individual foci. Quantification of γH2AX intensity for cells in each treatment group confirms that RT with magnetic microdiscs led to a significant increase in γH2AX density in T98G cells loaded with microdiscs. The results suggest that the magnetic microdiscs' intrinsic composition significantly enhances the radiation cytotoxicity in human GBM cells. Furthermore, the fluorescence quantification revealed an increased γH2AX staining level in GBM cells loaded with microdiscs in a dose-dependent manner; indeed Fig. 4 shows a higher H2AX staining for cells incubated with a concentration of 30 microdiscs per cell than cells incubated with 10 microdiscs per cells. Finally, GBM control cells with internalized microdiscs did not show an increase in the γH2AX expression in the absence of radiation. Such an observation confirms that the intracellular presence of magnetic microdiscs does not alter DNA in the absence of ionizing radiation.

### Simulations

The dose distributions around a microdisc were calculated by means of Monte Carlo simulations. The microdisc is immersed in water and placed 400 μm away from a parallel plane source, with the beam perpendicular to the plane of the microdisc. Models were selected for the water surrounding the microdisc, with the atomic de-excitation module activated.^20,21^ To replicate the irradiation conditions used experimentally, the spectrum of a 195 kV X-ray tube, with 0.8 mm beryllium inherent filter and 0.5 mm additional copper filtering was employed.

A dose enhancement is observed predominantly around the bases of the disc, rather than the sides. The dose distribution observed is mainly a factor of two components. Firstly, it is a result of the directionality of the electrons produced by the photon–microdisc interactions. Very few of the electrons generated will be scattered perpendicular to the beam direction; most of them will be generated towards the beam's direction. Secondly, the shape of the microdisc allows more electrons to escape towards the bases. This is a similar situation with the properties of some nanoparticles (NPs). Normally small NPs will allow more secondary electrons to escape.^22^ On the other hand, when the NPs aggregate, the secondary electrons from the inner NP are more likely to be reabsorbed, rendering the outermost NPs to contribute to the dose enhancement.^23^

**Fig. 7:**
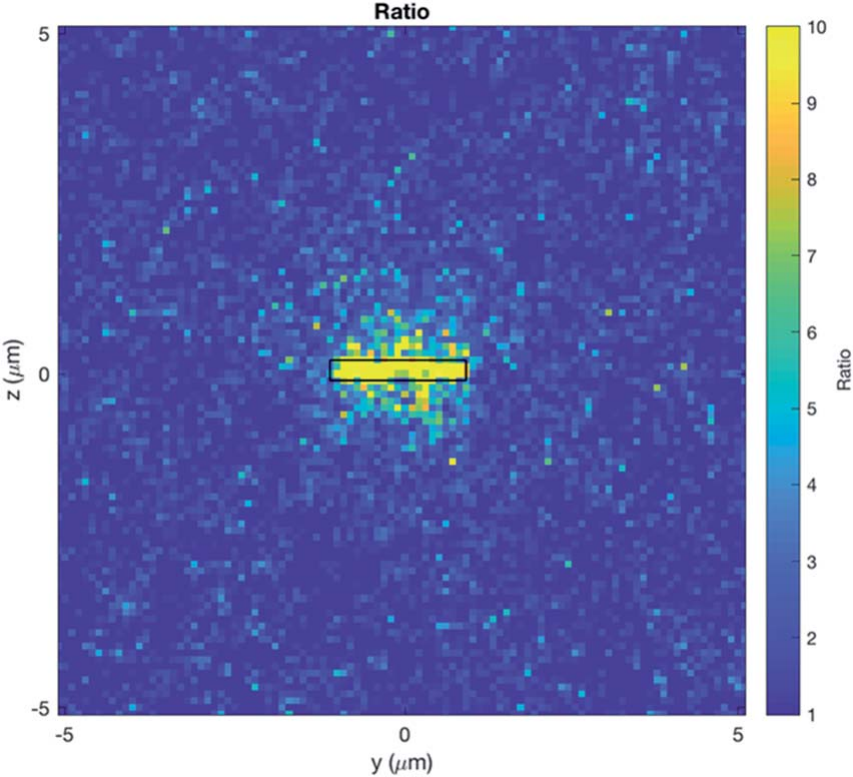
Ratio of the dose with to without the microdisc at the *YZ* plane.

In the Fig. 7, one can notice that the enhancement is localized at the very close vicinity of the discs, with a dose enhancement of approximately 5-fold observed within 0.5 μm around the microdisc, when the microdisc is perpendicular to the beam direction. This result is useful to provide explanations for our experimental results, particularly on the intracellular mechanisms involved in microdiscs-loaded T98G cells after irradiation.

### Combining radioenhancement with mechanical damage, cell survival assessment

We aimed to further investigate the use of every therapeutic facet of the engineered magnetic microdiscs, both in exerting mechanical torque when exposed to magnetic fields, and emitting secondary electrons when exposed to ionizing radiations. For this purpose, a cell survival assay was performed to correlate the overall induced cellular damage to the proliferation of the glioblastoma cells.

Compared to cells treated with RT alone with different radiation doses ranging from 1 Gy to 8 Gy (Group A), treated cells irradiated with similar doses exhibit a large decrease in the surviving fraction when microdiscs are loaded into cells prior to the radiations (Group B). The proliferation of the cells was even lower when the radiation therapy was followed by exposure to a rotating magnetic field (Group C).

**Fig. 8:**
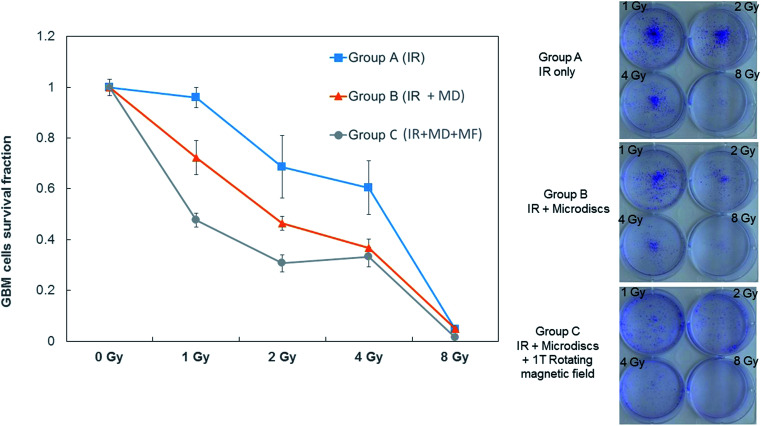
Survival fraction curves for T98G GBM cells submitted to three different treatments. (Group A) Irradiations only. (Group B) Irradiations in the presence of internalized microdiscs (MD). (Group C) Irradiations in the presence of internalized microdiscs and subsequently submitted to a 1 Tesla rotating magnetic field.

The survival rate shown in Fig. 8 exhibits a decreasing trend with increasing dose of X-rays, as expected. When T98G cells loaded with microdiscs are exposed to the same X-ray doses, the survival rates are lower than those of the control group (a) confirming the radioenhancement effect expected and corroborating the previous γH2AX staining results. As for Group (c) exposed to the rotating magnetic field, survival fractions are the lowest of all conditions, confirming that the magnetic disc-mediated radioenhancement combined with a further mechanical weakening through the disc rotation cause an effective reduction in the proliferation of cells.

## Discussion

To our knowledge, this is the first study that investigates the radioenhancement through heavy metal excitation combined with another physical phenomenon such as intracellular mechanical damage. The findings indicate that magnetic microdiscs play a synergistic role in enhancing glioblastoma cancer cell death during RT. In this work, we evaluated the influence of two functionalities of gold covered magnetic microdiscs: radioenhancement through their multi-layered intrinsic composition and mechanical disruption thanks to the magnetically driven mechanical torque they exert on cells. On the radioenhancement, findings revealed that the cellular internalization of these engineered nanomaterials led to a significant increase of DNA damage on glioblastoma cells after irradiation with X-rays, *in vitro*. Although the accelerators commonly used in radiotherapy deliver beam at the MeV range, we used for this study a low X-ray energy of 195 keV. There is an interest in using low energy X-ray sources that can be placed into the tumour cavity at the time of surgery for intra operative radiation therapy. This technology has gained a growing interest in the past two decades as a replacement to external beam radiation treatment.

As mentioned previously, rather than a blood systemic delivery, the magnetic microdiscs are directly put into the tumor cavity after the surgical resection. Different options may be used for the microdiscs delivery, we think of the microdialysis catheters as being the most suitable technique as microdialysis catheters are already used in brain tumors to deliver locally therapeutic agents. Then, the irradiation procedure will be very similar to the electron beam Inta Operative Radiation Therapy (IORT) where a low voltage energy source will be placed inside the tumor cavity to irradiate locally the tumor cavity, previously loaded with the microdiscs. A rotating magnetic field would be applied subsequently to actuate the magneto mechanical component of the discs in order to increase the cancer cells death. This technology allows to efficiently sterilize the cavity from remaining tumor cells and to bridge the therapeutic gap between surgery and radiotherapy. Some studies show evidence that radiation damage to mitochondria and to cell membrane may contribute also to the cytotoxic effect of radiation.^24^ Results revealed by our Monte Carlo simulations suggest that, unless the microdisc is touching the nucleus surface, direct DNA damage would not be expected. Rather, the concentrated energy deposited around the microdisc could lead to the increased production of reactive oxygen species (ROS) during RT that reaches the nucleus causing DNA structural damage.^25^ The ROS overproduction is also associated with the release of cytochrome C, and other apoptogenic factors.^26^ One can conclude that the observed radioenhancing effect may also be caused by the production of reactive oxygen species that lead to increased oxidative stress within the cell. These results are in line with other publications investigating nanostructures. For example, Laprise-Pelletier *et al.*^27^ showed that radioactive nanoparticles could lead to increased dose deposition around the NP, but the energy deposited is confined in a region close to the NP. In this case, the increased reactive oxygen species created from the dose enhancement are more likely to contribute to the radioenhancement effect observed. However, it is still commonly accepted that the most effective damage induced by radiotherapy is the induction of single and double-strand DNA breaks, with γH2AX foci being the sensors of DNA double-strand breaks. In the present study, we demonstrated that microdisc driven radioenhancement induces a high level of γH2AX foci staining when compared to RT treatment alone. In accordance with the DNA damage results, the cell survival fraction assay revealed that the proliferation rate was drastically affected by the microdisc mediated radioenhancement. The survival fraction was even more affected when the radioenhancement was combined with the exposure to the rotating magnetic field, thus combined with the internal mechanical weakening of cells.

This perspective may also be true for other complex magnetic particles that may have a possible radioenhancement effect, and which should be tested in the future, such as iron–nickel alloy vortex type particles.^11^ More importantly, studies on the mechanism inducing radioenhancement indicated that different shaped gold nanostructures affected the radioenhancement effect through regulating the ROS level, with the higher sensitization enhancement ratios directly linked to the amount of gold nanomaterial cellular uptake.^9^

We believe this study brings a new insight into the field of radioenhancement mediated by internalized metallic materials. With most of the studies to date focusing on nano-sized materials, this is the first study that reports the impact of micron-sized materials with high aspect ratio. Regarding the uptake efficiency, Champion and coworkers^28^ previously reported that elongated particles with higher aspect ratio are more prone to phagocytosis. In the case presented here, internalization is enhanced due to the large surface area of the magnetic discs, which increases adhesion to cells. This increase in adhesion contact area will also enhance the interaction between the flat disk-shaped particle and the membrane, facilitating full wrapping through membrane bending. The adhesive interactions between the flat discs and the membranes seem to be sufficiently strong to compensate for the energetic cost of membrane bending.^29^

While the ease of internalization of such particles by cancer cells is an advantage for the treatment modalities presented above, one cannot deny the possibility of side effects if such particles are internalized by healthy cells. Thus an extensive study on diffusing the microdiscs in a 3D environment and targeting a specific cell subpopulation is critical. Several targeting strategies include the covalent grafting of antibodies on the gold outer layers covering the magnetic discs. This ligand–receptor mediated targeting strategy involves the construction of self-assembled monolayers of thiols on the gold surface.^13^ It is well established that cancer cells overexpress particular membrane receptors. For example, vascular endothelial growth factor receptor (VEGFR), transferrin receptors, integrins, or folate receptor have been widely exploited for tumor targeting strategies. In practice, tumors exhibit particular biological and physico-chemical properties that need to be exploited to optimize the targeted biodistribution of the microdisc *in vivo* (*e.g.* local blood flow, pH condition, vasculature and extracellular matrix organization). This novel mechanism of cell kill based on direct DNA damage from intensely ionising particle, and physical disruption of tumour cells, may be attractive in being independent of any specific mutation pathways, which may help overcome treatment resistance in highly heterogenous tumours such as GBM.

It is understandable that concerns on the fate of the magnetic microdiscs after the treatment might be raised. However, median survival for the group of patients diagnosed with glioblastoma multiforme is 12–15 months even with aggressive treatment. We believe that concerns about long term toxicity due to particles accumulation in the brain need to be balanced against the poor long-term survival rates.

If the magnetic microdiscs developed in this study were to demonstrate very high levels of toxicity to normal brain parenchyma in animal models, then alternative routes exist. Clinicians have the capability to instill the magnetic microdiscs, magnetically activate them at the time of operation, and then wash out particles from the tumor cavity. Another possibility for the clearance of any remaining microdiscs which relies on magnetic attraction forces could also be explored. After the treatment, the microdiscs magnetic component will be exploited in order to attract them toward a high magnetic gradient source. Such a source might be a catheter-like device coated with a high remanence magnetic material that attracts the surrounding particles present in the sterilized cavity after the treatment.

It should be considered that there is a wealth of research on the feasibility of localized delivery of nanoparticles bound to a range of therapeutic agents in humans. Mechanisms include stereotactic injection, implantation into the surgical cavity at the time of surgery, or using convection enhanced delivery *via* microdialysis catheters. In each case, systemic toxicity from the payload has been demonstrated to be minimal.^30^

## Material and methods

### Cells culture

The human glioma cell line T98G was purchased from the American Type Culture Collection (Manassas, VA., USA) and cultured at 37 °C in a 5% CO_2_ atmosphere in Roswell Park Memorial Institute (RPMI) (1640) medium containing 2% penicillin and streptomycin antibiotic and 10% fetal bovine serum.

### Magnetic microdiscs fabrication

The microdiscs consist of pairs of 0.9 nm thick CoFeB magnetic layers which are antiferromagnetically coupled *via* Ruderman–Kittel–Kasuya–Yosida (RKKY) interactions through a Pt (0.4 nm)/Ru (0.9 nm)/Pt (0.4 nm) spacer layer. Each pair of magnetic layers is grown on tantalum/platinum buffer Ta (2 nm)/Pt (2 nm) with a 2 nm Pt cap. This construct is then repeated 10 times. The microdiscs are capped top and bottom with 10 nm of Au as shown in Fig. 1. The total stack is: Au (10 nm)/[Ta (2 nm)/Pt (2 nm)/CoFeB (0.9 nm)/Pt (0.5 nm)/Ru (0.9 nm)/Pt (0.5 nm)/CoFeB (0.9 nm)/Pt (2 nm)]10/Au (10 nm). This multilayer stack is grown using DC magnetron sputtering onto UV lithographically patterned pillars of MA-N 1410 photoresist. The photoresist pillars are then dissolved in acetone, releasing the 2 micron wide discs into solution.

### Magnetic field generator

The rotating magnetic field station used a NdFeB Halbach Array (Bunting Magnetics Europe Ltd., Hertfordshire, UK), mounted on a motor that controls its rotation. The magnet produces a uniform 1 Tesla magnetic field diametrically across the central air gap. Cells were placed inside the 2 cm wide central cylindrical air gap.

### Magnetic microdiscs internalization

3 × 10^4^ T98G cells were seeded on glass coverslips previously placed into 12 well plate. 6 × 10^5^ magnetic microdiscs were added into each well and incubated with the cells for 24 hours. Post incubation, the cells were washed and fixed with 4% paraformaldehyde in PBS buffer for 15 minutes. After washing in PBS buffer, the glass coverslips were metalized by sputtering a 2 nm layer of tantalum. Overall the sample is composed by cells grown on glass coverslips, fixed on silicon substrate.

**Fig. 9:**
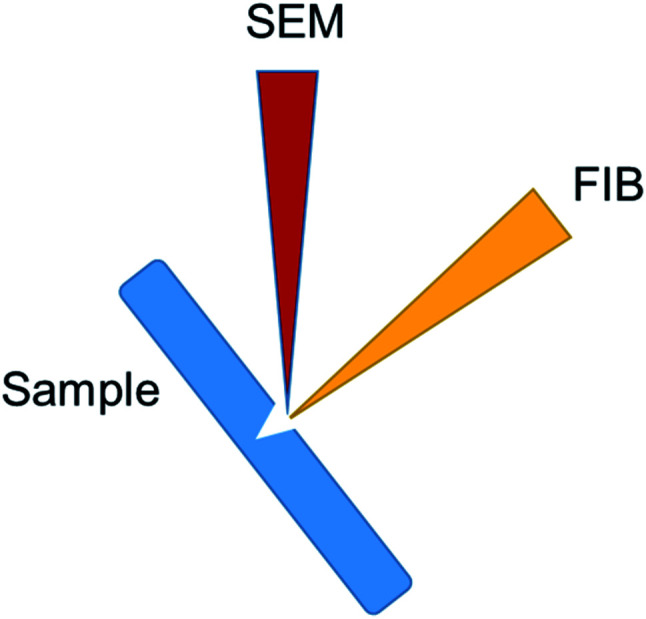
Schematic showing the positioning of the sample, FIB and SEM beams during FIB-SEM cross sectioning.

Before the FIB milling, a thin layer on platinum (1 μm) is deposited on the surface of the region of interest, to ensure a sharp edge of the sample surface. For the FIB sectioning, the sample is positioned at a 52° tilt, facing the FIB beam. To ensure that the FIB beam is at a right angle to the sample surface in order to cut a precise trench on the site of interest. As the sample is positioned at a junction between the FIB and SEM beams, SEM imaging is carried out both after and during the FIB milling. In order to increase the edge sharpness of the cells, 1 μm thin layer of platinum is deposited *in situ* at the surface on the regions of interest. Fig. 9 represent a schematic showing the positioning of the sample, FIB and SEM beams during FIB-SEM cross sectioning.

### Magneto-mechanically induced damage

96-well plates were previously cut into 1.5 cm wide strips to allow their insertion into the central air gap of the rotating magnet. After sterilization of the strips, glioma cells were seeded into the wells at 8000 cells per well, with three replicates for each condition. The cells were incubated with magnetic microdiscs for 24 hours. Post incubation, cells were put into the center of the magnetic field and treated for 20 minutes. After treatment, cells were rinsed in PBS and incubated 30 minutes at room temperature with 4 μM Ethidium homodimer-1 staining (LIVE/DEAD Cell Viability Assays, Thermo Fisher). Cells were rinsed once with PBS prior to imaging with Leica TCS SP5 confocal microscope.

### Cytotoxicity assays

The cytotoxicity of the magnetic microdiscs was assessed by the standard 3-(4,5-dimethylthiazol-2-yl)-2,5-diphenyltetrazolium bromide (MTT) assay (Abcam ab211091, Cambridge, UK). T98G cells were plated into 96 well plates (8000 cells per well) for 24 h. Then different concentrations of microdiscs (10, 20, 30, 40 and 50 ND per cell) were added, the cells were cultured for another day.

After 24 h incubation, MTT solution was added into each well and cells were cultured with MTT at 37 °C for 4 h. After removal of the MTT solution, 100 μL of dimethyl sulfoxide (DMSO), was used to dissolve the formed formazan crystals. The relative absorbance at 570 nm was read on the Omega microplate reader. 3 replicates were included for each condition and the experiments were repeated twice. In addition, the scattering effects due to the magnetic microdiscs were accounted for by including the microdiscs in the control group.

### Irradiation procedure

Samples were irradiated using the XStrahl RS225 research cabinet (Xstrahl Limited, Surrey, UK) using the standard settings (195 kV, 10 mA). The irradiation doses varied between 0 and 16 Grey. Cell culture dishes were irradiated horizontally in the upright position with the lid kept on the culture dishes.

### DNA damage detection

T98G cells were incubated with microdiscs for 24 h and then exposed to 1, 2, 4, 6, 8 and 16 Gy X-rays. The supernatant was removed 1 h approximately after radiations. T98G cells were immersed in 4% paraformaldehyde for fixation for 15 min and then washed with PBS three times. After that, the cells were treated with Triton X-100 for 10 min to damage their membranes. Then, the cells were soaked in a blocking buffer (1% bovine serum albumin, 22.5 mg ml^−1^ glycine in PBS solution) for 1 h and incubated with antihistone γH2AX rabbit polyclonal antibodies (ab11174, Abcam, Cambridge, UK) (diluted 1 : 1000 with PBS) overnight at 4 °C, followed by washing with PBS three times. The cells were further incubated with anti-rabbit secondary antibody (diluted 1 : 2000 with PBS) for 2 h in the dark and then washed with PBS three times. Stained cells were observed with the confocal microscope (Leica confocal).

### Cell proliferation assay

Controls include the following conditions: bare cells (without microdiscs), not irradiated, and cells loaded with microdiscs, not irradiated.

Treated cells were irradiated with different doses ranging from 1 Gy to 8 Gy. To facilitate the results reading, conditions were divided into three groups; Group (a) are cells treated with irradiations only. Group (b) represents cells loaded with magnetic microdiscs (30 units per cell) and treated with the same radiation doses as cells in group (a). Group (c) are cells loaded with magnetic microdiscs, treated with the same radiation doses as cells in group (a and b) and submitted to 20 min of rotating 1 T magnetic field.

After 24 hours incubation time with the microdiscs to allow their internalization, groups (a), (b) and (c) received the following radiation doses of 1, 2, 4, and 8 Gy. When all treatments were completed, the cells were trypsinized to allow their detachment from the culture dishes. The cells detachment process was carefully monitored to ensure that the entire cells population has been harvested from the well plate, for all the samples.

The collected cells were resuspended in fresh complete medium, seeded into 6 wells plates, and left in the incubator for 8 days, allowing enough time for their proliferation. For visualization convenience, photographs of wells seeded with 1000 cells each were used.

Cells were fixed with 4% paraformaldehyde solution and stained for 4 hours with 0.5% crystal violet solution.

As for counting the survival fractions, we used lower cell densities of 200 cells per well. Results shown in Fig. 5 reveal a marked decrease of irradiated microdiscs-T98G cells proliferation compared to T98G cells receiving irradiation only. Group (c) samples exposed to the rotating magnetic field, therefore receiving the mechanical disruptive treatment, exhibited an even lower proliferation rate than group (a) and (b).

The cell survival fraction of each group was calculated by the ratio of the number of colonies formed by seeded cells after various treatments normalized to the untreated cells. The results of differences in survival fractions of the three groups are shown in Fig. 5(a).

Note that for each group there were three replicates.

## Simulations

Simulation details are available in the ESI section.†

## Conflicts of interest

The authors declare no competing interests.

## Supplementary Material

RA-010-D0RA00164C-s001
