# Supplementary material for: Versatile magnetic microdiscs for the radio enhancement and mechanical disruption of glioblastoma cancer cells
Source: RSC Adv. 2020 Feb 25;10(14):8161–71. doi: 10.1039/d0ra00164c (PMC9092955; doi:10.1039/d0ra00164c)
Supplement: RA-010-D0RA00164C-s001 [file RA-010-D0RA00164C-s001.pdf]

Electronic Supplementary Material (ESI) for RSC Advances.
This journal is © The Royal Society of Chemistry 2020

Versatile magnetic microdiscs for the radio enhancement and mechanical disruption of glioblastoma cancer cells

Selma Leulmi-Pichot [1], Sabrina Bentouati [1], Saif S Ahmad [2], Marios Sotiropoulos [3], Rajesh Jena[2] & Russell Cowburn[1]*

1- Department Of Physics, University of Cambridge, JJ Thomson Avenue, Cambridge CB3 0HE, United Kingdom.

2- Department of Oncology, University of Cambridge. Hutchison/MRC Research Centre, Cambridge Biomedical Campus, Cambridge CB2 0XZ, United Kingdom.

3- Division of Molecular and Clinical Cancer Sciences, School of Medical Sciences, Faculty of Biology, Medicine and Health. The University of Manchester, Manchester, United Kingdom.

Corresponding author: sl766@cam.ac.uk

Magnetic microdiscs internalization

The location of the magnetic microdiscs inside T98G cells was assessed using focused ion beam – scanning electron (FIB-SEM) based tomography. After their fixation and metallization, T98G cells incubated 24h with magnetic microdiscs are randomly selected and then milled using the ion beam and imaged using the electron beam creating a series of images of the interior of the cell. Internalized magnetic microdiscs are shown in Figure 1 and Figure 2.

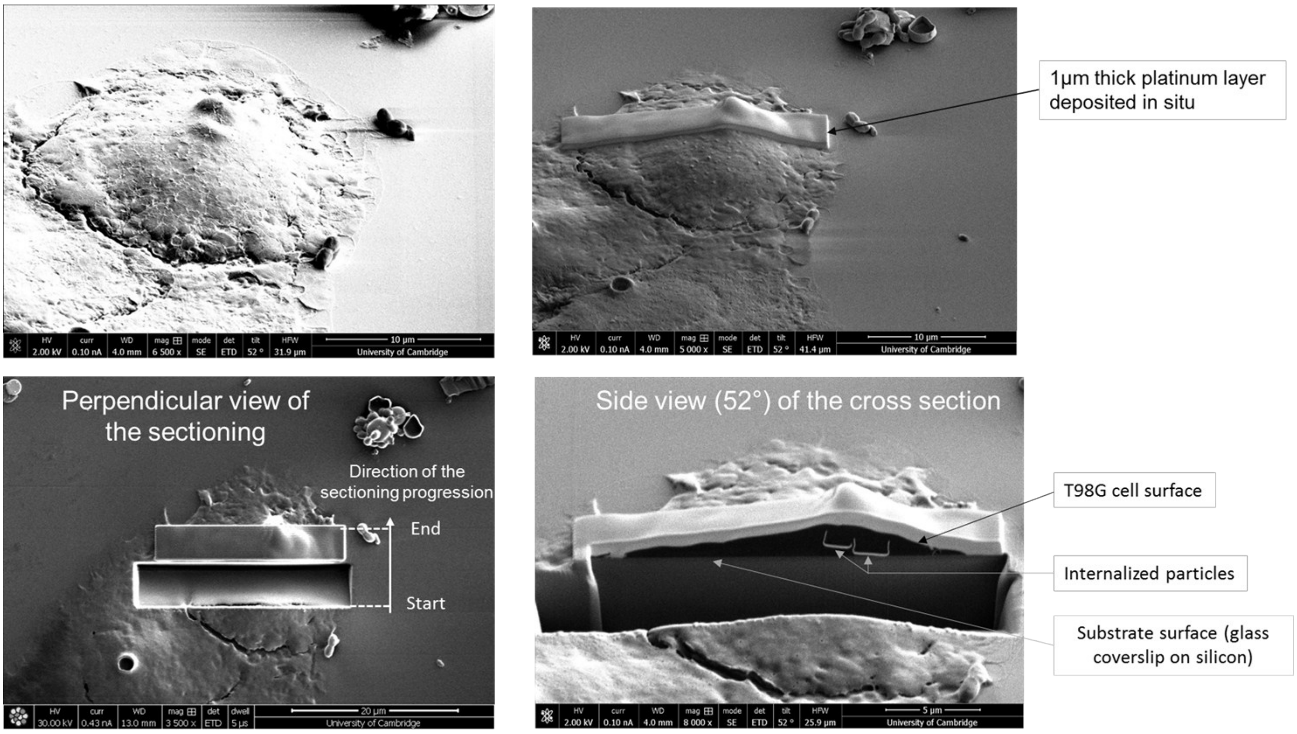

Figure 1 T98G cell cross sectionning and imaging with FIB-SEM

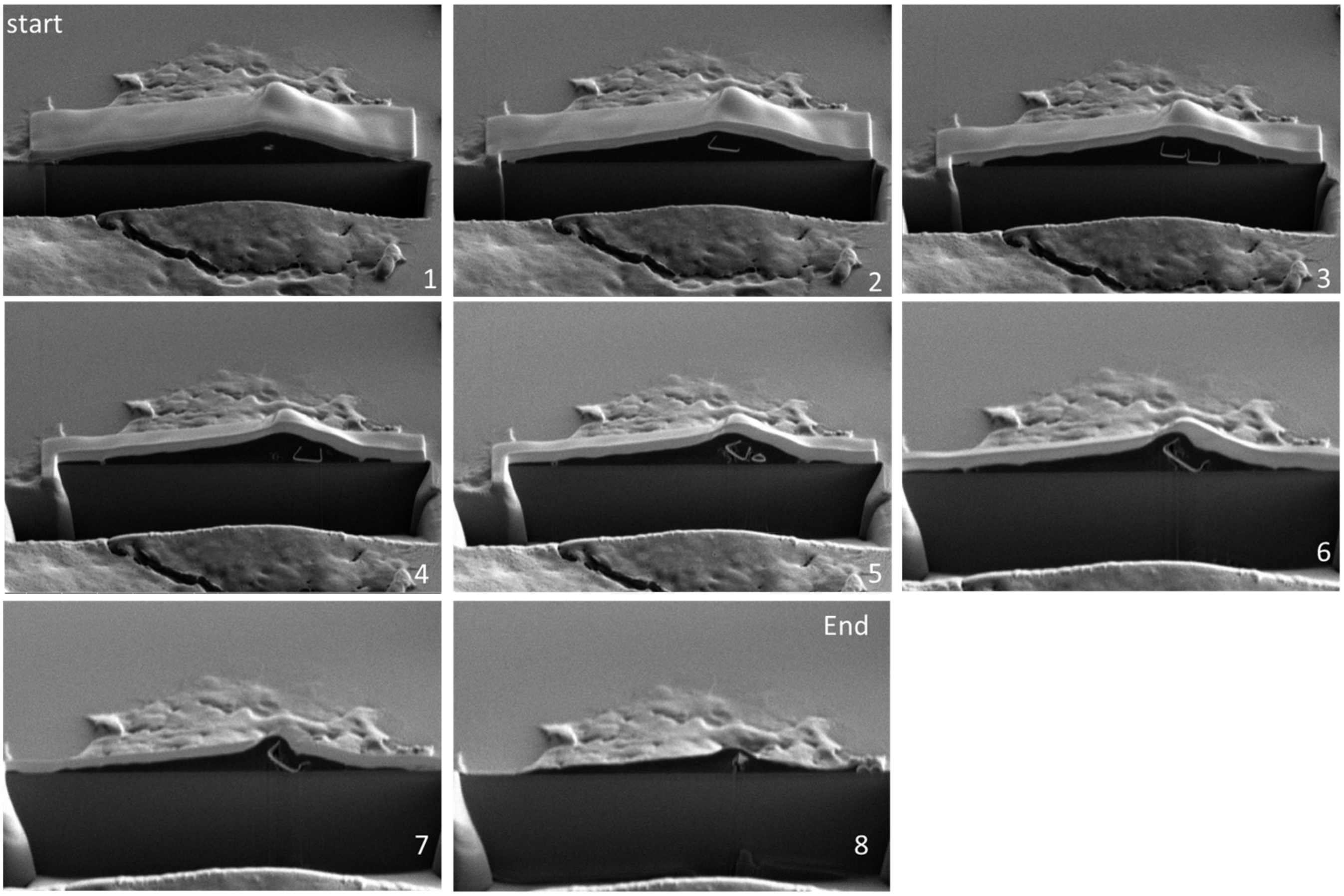

Figure 2 Full sectionning of the region of interest. The sectionning reveals the presence of internalized microdiscs within the selected region.

Dosimetric characterisation of an irradiated antiferromagnetic nanodisc

1. Simulations

1.1. The antiferromagnetic nanodisc

The antiferromagnetic nanodisc investigated in this study has a diameter of 2 μm and thickness of 132 nm. It consists of an outer layer of 10 nm of gold and inside the following group of materials is repeated 10 times: Pt (2 nm), Ta (2nm), CoFeB (0.9 nm), Pt (0.25 nm), Ru (0.9 nm), Pt (0.25nm), CoFeB (0.9 nm), Pt (2 nm), Ta (2 nm), as demonstrated in Figure 3.

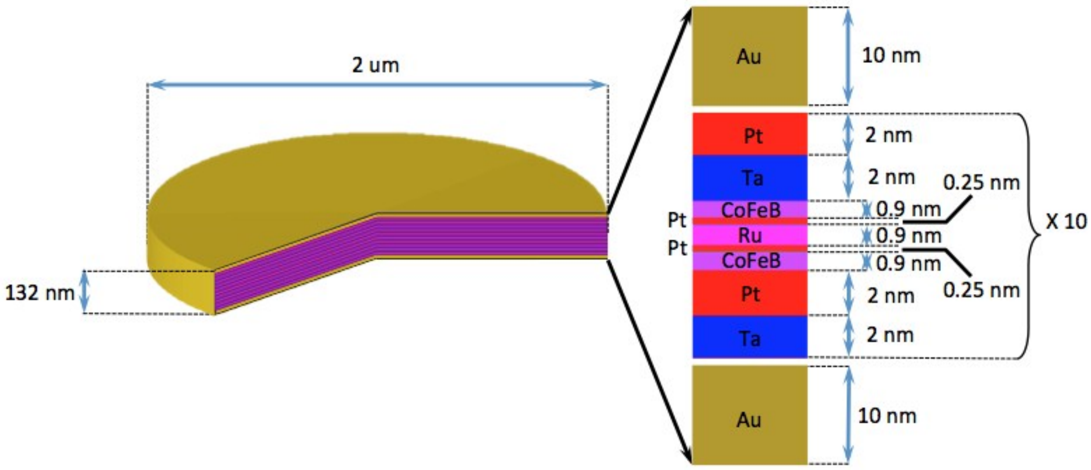

Figure 3. Simulation model of the material composition of the antiferromagnetic nanodisc.

1.2. Simulation details

The model of the nanodisc is imported in the Geant4.10.4 simulation toolkit. The nanodisc is placed 400 μm away from a parallel plane source (Figure 4), immersed in water. The Livermore interaction models are selected for the nanodisc, while the Geant4-DNA (Incerti et al. 2010A; Incerti et al. 2010B; Bernal et al. 2015; Incerti et al. 2018) models were selected for the water surrounding the nanodisc, with the atomic de-excitation module activated, as in our previous publications (Sotiropoulos et al. 2017A; Sotiropoulos et al. 2017B). The dose scoring is done through two voxelized mesh dose scorers, 40 μm x 40 μm x 1 μm and 1 μm x 40 μm x 40 μm with voxel size of 0.1 x 0.1 x 1 μm^3 and 1 x 0.1 x 0.1 μm^3 for the parallel and perpendicular scoring mesh respectively. The simulations were run for 4 billion initial photons.

1.3. X-ray spectrum

To replicate the irradiation conditions typically used in a clonogenic survival assay, the spectrum of a 195 kV x-ray tube, with 0.8 mm Be inherent filter and 0.5 mm additional Cu filtering was employed. The spectrum was generated from the SpekCalc

program (Poludniowski & Evans 2007; Poludniowski 2007). The generated spectrum is shown in Figure 5.

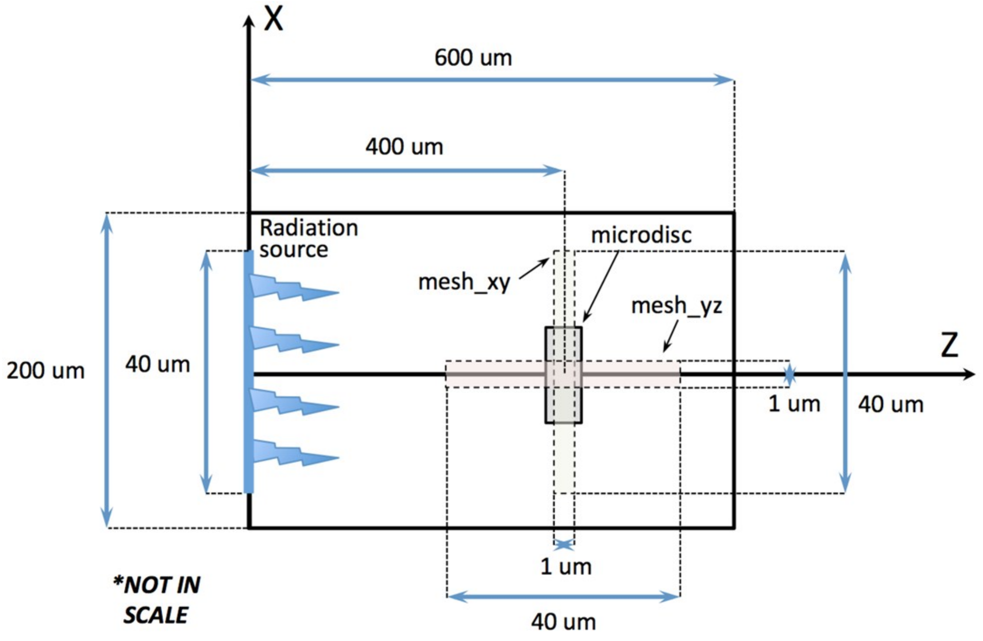

Figure 4. Irradiation geometry for the dosimetric characterization of the nanodisc. The nanodisc axis is parallel to the Z direction. The dose is scored into two voxelized mesh scorers, parallel and perpendicular to the nanodisc, mesh_xy and mesh_yz respectively.

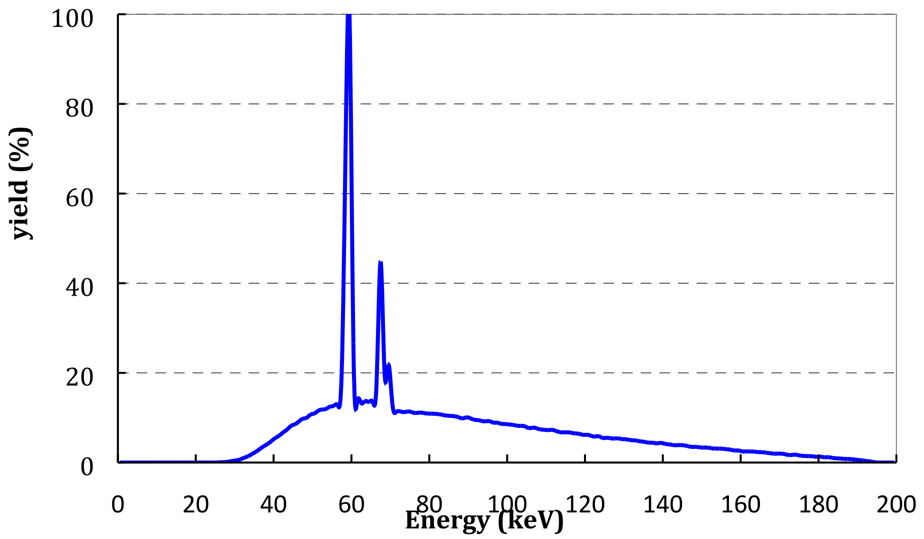

Figure 5. X-ray spectrum at 195 kV generated by the SpekCalc program, with 0.8 mm inherent Beryllium filter and 0.5 mm of additional Cooper filter.

2. Results

The ratio and difference of the dose with the nanodisc to the dose without the nanodisc at the XY and YZ planes is shown in Figures 6-9.

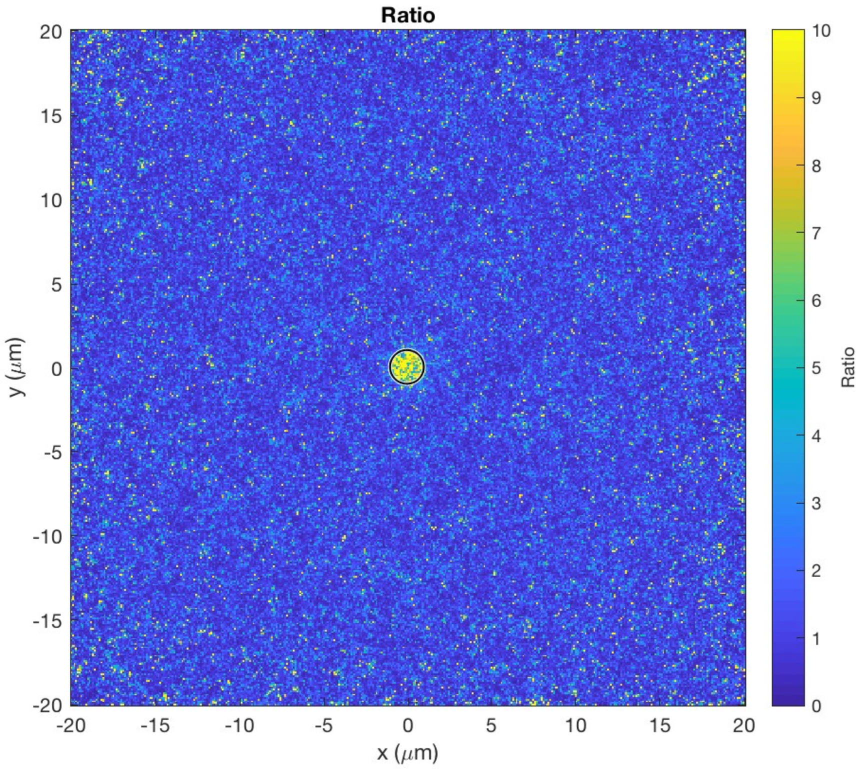

Figure 6. Ratio of the dose with to without the nanodisc at the XY plane.

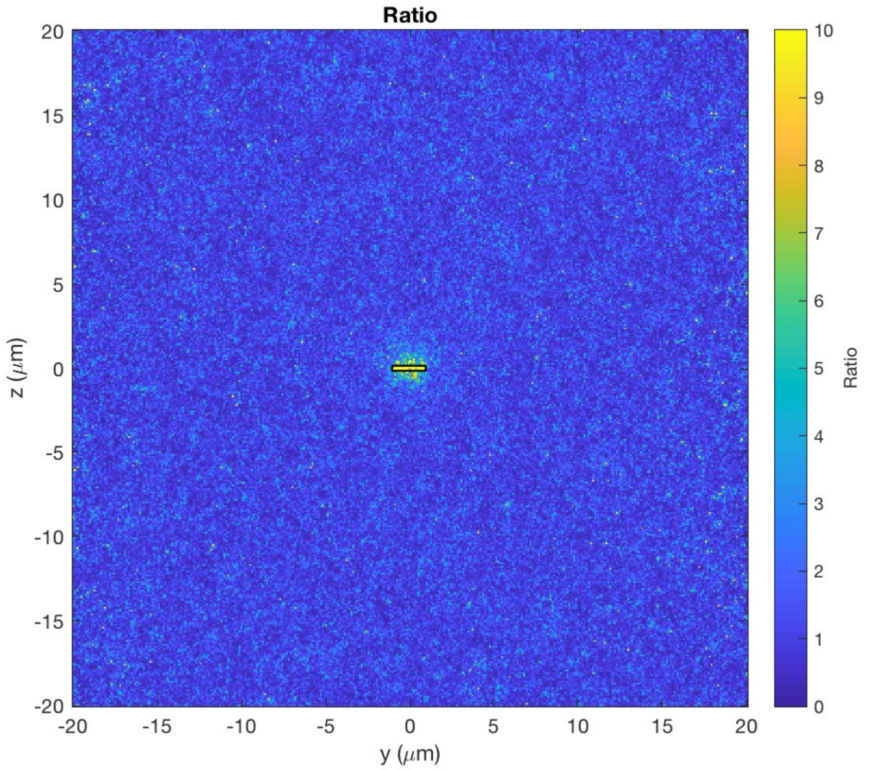

Figure7. Ratio of the dose with to without the nanodisc at the YZ plane.

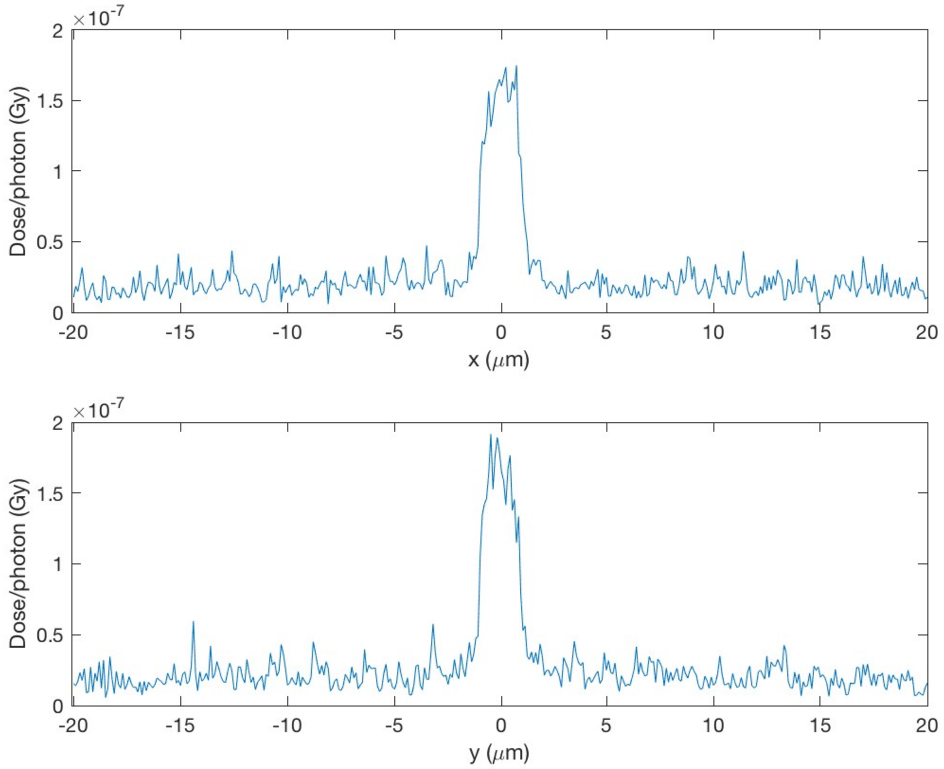

Figure 8. Dose profiles along the X and Y axes respectively.

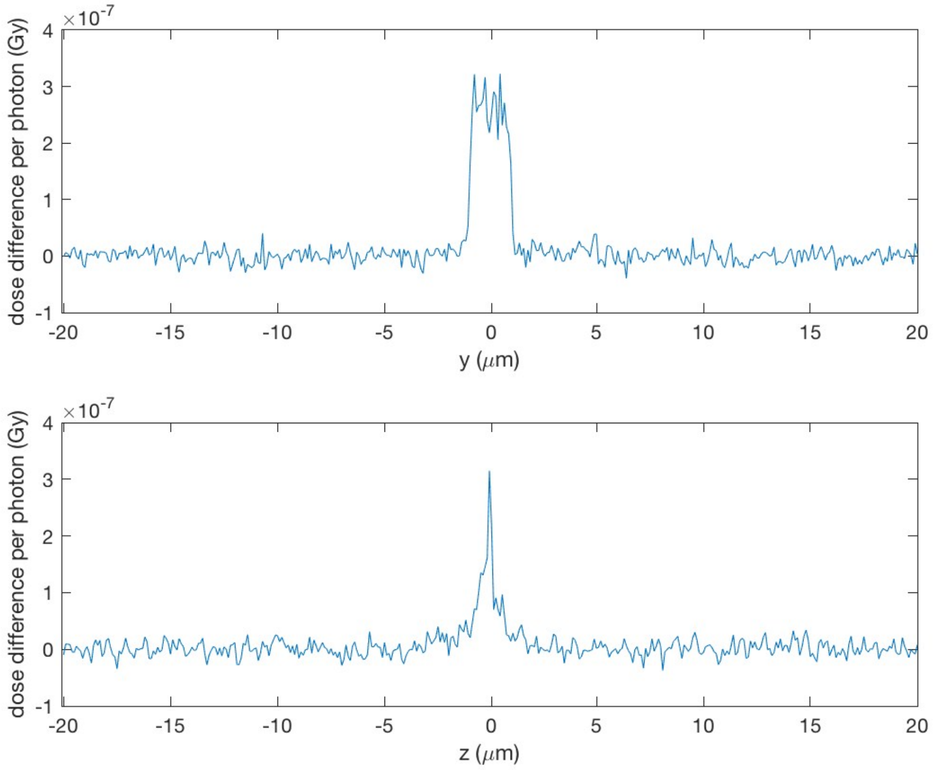

Figure 9. Profiles of the dose difference along the Y and Z axes respectively.

3. Discussion

The dose distributions around a nanodisc were calculated by means of Monte Carlo simulations. A dose enhancement is observed predominantly around the bases of the disc, rather than the sides. The dose distribution observed is mainly a factor of two components. Firstly, it is a result of the directionality of the electrons produced by the photon-nanodisc interactions. Very few of the electrons generated will be scattered perpendicular to the beam direction; most of them will be generated towards the beam's direction. Secondly, the shape the nanodisc allows more electrons to escape towards the bases. This is a similar situation with the properties of some NPs. Normally small NPs will allow more secondary electrons to escape (Lechtman et al. 2011). On the other hand, when the NPs aggregate the secondary electrons from the inner NP are more likely to reabsorbed, rendering the outermost NPs to contribute to the dose enhancement (Kirkby et al. 2017). In addition, the gold layer surrounding the nanodisc can absorb some of the low energy electrons produced by the innermost layers of the nanodisc.

As a radiosensitization has been observed in vitro when the antiferromagnetic nanodiscs are internalised into cells, it would be interesting to speculate how this dose enhancement might contribute to the radiosensitization effect. Unless the nanodisc is touching the nucleus surface, a direct DNA damage wouldn't be expected. Rather, the concentrated energy deposited around the nanodisc could lead to the increased production of reactive oxygen species, with the

potential to diffuse to the nucleus and attack the DNA molecule. Furthermore, the produced reactive oxygen species could lead to increased oxidative stress in the cell. These results are in line with other publications investigating nanostructures. For example, Laprise-Pelletier et al. (2018) showed that radioactive nanoparticles could lead to increased dose deposition around the NP, but the energy deposited is confined in a region close to the NP. In this case, the increased reactive oxygen species created from the dose enhancement are more likely to contribute to the radiosensitization effect observed.

4. MTT cell cytotoxicity test

To ensure that the internalization of the microdiscs does not cause cell damage in itself, a cytotoxicity evaluation test was performed. Cell metabolic ability was examined by MTT assay. T98G cells were incubated with different concentrations ranging from 10 microdiscs/cell to 50 microdiscs/cell. Cells loaded with particles were compared to control cells. T98g cells loaded with microdiscs exhibited a decreased metabolism of tetrazolium salts in a dose-dependent manner. However, it is important to note that the internalized microdiscs did not affect the cells' viability. Live/Dead assay results suggest that the cells remain viable despite exposure to concentrations up to 50 particles per cell. The figure below show the results of different incubation times with the microdiscs (24h, 48h and 72h)

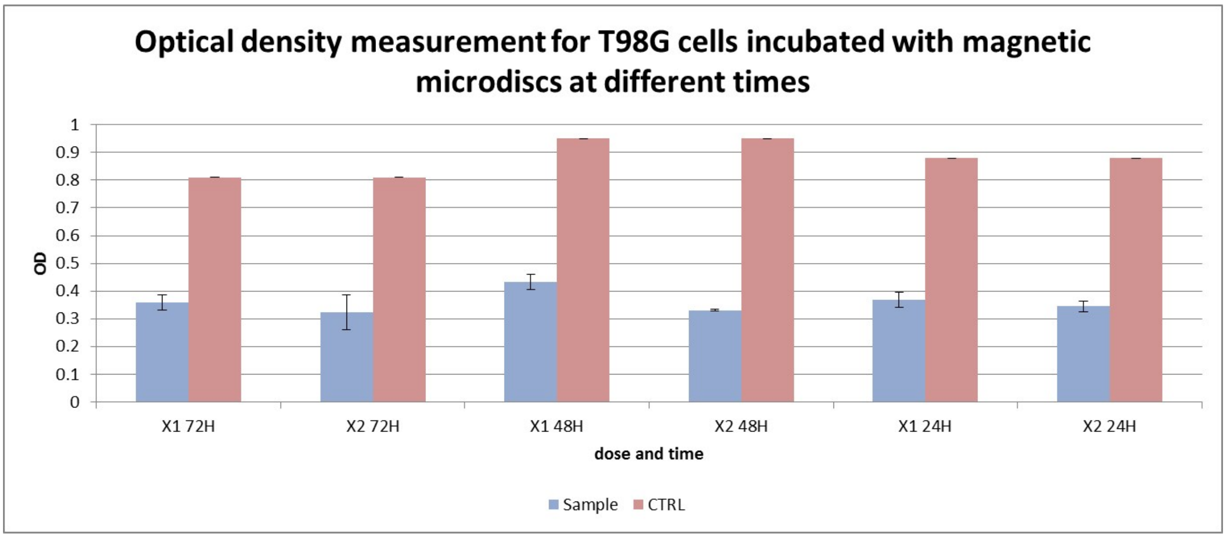

Figure 10 optical density measurement for T98G cells incubated with magnetic particles at three time points.

5. References

Bernal, M.A. et al., 2015. Track structure modeling in liquid water: A review of the Geant4-DNA very low energy extension of the Geant4 Monte Carlo simulation toolkit. *Physica Medica*, 31, pp.1–14.

Incerti, S. et al., 2010A. Comparison of GEANT4 very low energy cross section models with experimental data in water. *Medical physics*, 37(9), pp.4692–4708.

Incerti, S. et al., 2018. Geant4-DNA example applications for track structure simulations in liquid water: A report from the Geant4-DNA Project. *Medical Physics*, 45(8), pp.e722–e739.

Incerti, S. et al., 2010B. THE GEANT4-DNA PROJECT. *International Journal of Modeling, Simulation, and Scientific Computing*, 01(02), pp.157–178.

Kirkby, C. et al., 2017. Dosimetric consequences of gold nanoparticle clustering during photon irradiation: *Medical Physics*, 44(12), pp.6560–6569.

Laprise-Pelletier, M. et al., 2018. Intratumoral Injection of Low-Energy Photon-Emitting Gold Nanoparticles: A Microdosimetric Monte Carlo-Based Model. *ACS Nano*, 12(3), pp.2482–2497.

Lechtman, E. et al., 2011. Implications on clinical scenario of gold nanoparticle radiosensitization in regards to photon energy, nanoparticle size, concentration and location. *Physics in Medicine and Biology*, 56(15), pp.4631–4647.

Poludniowski, G.G., 2007. Calculation of x-ray spectra emerging from an x-ray tube. Part II. X-ray production and filtration in x-ray targets. *Medical Physics*, 34(6), pp.2175–2186.

Poludniowski, G.G. & Evans, P.M., 2007. Calculation of x-ray spectra emerging from an x-ray tube. Part I. Electron penetration characteristics in x-ray targets. *Medical Physics*, 34(6), pp.2164–2174.

Sotiropoulos, M. et al., 2017A. Geant4 interaction model comparison for dose deposition from gold nanoparticles under proton irradiation. *Biomedical Physics & Engineering Express*, 3(2), p.025025.

Sotiropoulos, M. et al., 2017B. Modelling direct DNA damage for gold nanoparticle enhanced proton therapy. *Nanoscale*, 9(46), pp.18413–18422.
